# Microbial Fuel Cell Construction Features and Application for Sustainable Wastewater Treatment

**DOI:** 10.3390/membranes13050490

**Published:** 2023-04-30

**Authors:** Hridoy Roy, Tanzim Ur Rahman, Nishat Tasnim, Jannatul Arju, Md. Mustafa Rafid, Md. Reazul Islam, Md. Nahid Pervez, Yingjie Cai, Vincenzo Naddeo, Md. Shahinoor Islam

**Affiliations:** 1 Department of Chemical Engineering, Bangladesh University of Engineering and Technology, Dhaka 1000, Bangladesh; hridoyroy@che.buet.ac.bd (H.R.);; 2 Department of Civil Engineering, Louisiana Tech University, Ruston, LA 71270, USA; 3 Sanitary Environmental Engineering Division (SEED), Department of Civil Engineering, University of Salerno, via Giovanni Paolo II 132, 84084 Fisciano, SA, Italy; 4 Hubei Provincial Engineering Laboratory for Clean Production and High Value Utilization of Bio-Based Textile Materials, Wuhan Textile University, Wuhan 430200, China; 5 Department of Textile Engineering, Daffodil International University, Dhaka 1341, Bangladesh

**Keywords:** MFCs, construction features, membrane, sustainable, wastewater

## Abstract

A microbial fuel cell (MFC) is a system that can generate electricity by harnessing microorganisms’ metabolic activity. MFCs can be used in wastewater treatment plants since they can convert the organic matter in wastewater into electricity while also removing pollutants. The microorganisms in the anode electrode oxidize the organic matter, breaking down pollutants and generating electrons that flow through an electrical circuit to the cathode compartment. This process also generates clean water as a byproduct, which can be reused or released back into the environment. MFCs offer a more energy-efficient alternative to traditional wastewater treatment plants, as they can generate electricity from the organic matter in wastewater, offsetting the energy needs of the treatment plants. The energy requirements of conventional wastewater treatment plants can add to the overall cost of the treatment process and contribute to greenhouse gas emissions. MFCs in wastewater treatment plants can increase sustainability in wastewater treatment processes by increasing energy efficiency and reducing operational cost and greenhouse gas emissions. However, the build-up to the commercial-scale still needs a lot of study, as MFC research is still in its early stages. This study thoroughly describes the principles underlying MFCs, including their fundamental structure and types, construction materials and membrane, working mechanism, and significant process elements influencing their effectiveness in the workplace. The application of this technology in sustainable wastewater treatment, as well as the challenges involved in its widespread adoption, are discussed in this study.

## 1. Introduction

In the decades ahead, the world is approaching a crisis regarding energy and the environment. Hence, sustainable development is the way ahead to ensure environmental safety and maximize the utilization of renewable sources of energy. The energy and environmental catastrophe in terms of numerous pollutions and carbon emissions manifest the urgency for a transition towards renewable energy to abate the total dependence on fossil fuels, bending global warming trends, and addressing the descending level of existing sources. Water pollution is a great concern in terms of maintaining environmental sustainability [1,2].

With rapid industrialization, water bodies are becoming contaminated with significant amounts of organic pollutants, heavy metals, and other toxic components. Although industrialization is required for economic and social growth, its negative impact on the environment through several processes that emit harmful pollutants should be taken into consideration. The discharge of inadequately treated wastewater can have a detrimental effect on ecology and bio-diversity [3]. Therefore, treatment methods in harmony with green sustainable development are needed to propel the impulse of industrialization to sustain the shield against pollution for water reclamation and reuse. In the present energy crisis scenario, conventional wastewater treatment plants are no longer suitable since they are the primary energy sinks. The conventional wastewater treatment process is expensive and energy intensive. These processes are also quite ineffective in removing refractory organic matters, heavy metals, and other persistent pollutants [4]. Not only that, some of the treatment methods require regeneration, which again produces hazardous wastes [5]. As a result, microbial fuel cells (MFCs) have emerged as a sustainable treatment technology capable of removing persistent organic pollutants and heavy metals [6,7,8]. MFCs are preferable to other advanced treatment technologies due their lower energy requirement and minimization of chemical usage [9]. Other than lower energy requirements, MFCs can also offer ample amounts of bioenergy to meet the energy crisis, opening a greener threshold for generating renewable energy with a view to achieving sustainable development and environmental safety.

A microbial fuel cell (MFC) degrades pollutants via anaerobic oxidation using microbes, e.g., algae, bacteria, etc., generating electrons in the process. These electrons passing through the external circuit, which allows electricity generation. The oxidation of the organic wastes is driven by the anaerobic respiration and metabolism of electrochemically active bacteria [10]. On the other hand, the electron passing through the circuit reaches the cathode, where it is accepted using an electron acceptor (O_2_ commonly used). MFCs can be termed as a fuel cell that converts the chemical energy of organics to electrical energy where microorganisms act as the biocatalysts [11]. The main advantage of MFCs compared with conventional low temperature fuel cells is their utilization of complex organic contaminants as fuel at the anode chamber. The operation of MFCs is performed at ambient temperature under neutral pH conditions. The exo-electrogenic bacteria can take part in the initiation of oxidation of contaminants with comparatively lower voltage applications [12]. 

Despite having potential to be sustainable technology, there remains several challenges in the implementation of MFCs for extracting energy from pollutants. The limitations are associated with their low power output, slow start-up, higher expense, and lower efficiencies compared with other technologies. A major obstacle to industrial implementation of this process is its inability to produce energy at a sufficient rate to satisfy a significant fraction of the energy requirements of a large-scale wastewater treatment facility. Further research and efforts are required to overcome the challenges associated with the system. There is also lack of understanding relating to the biology of the process. The mechanism of electron transfer of bacteria requires further investigation. It is required to produce more genetically modified microorganisms that can accelerate electron transfer, producing more energy. With further efforts to overcome the challenges associated with the process and technological advances, MFCs can be implemented as a sustainable and green technology on a commercial-scale, producing energy and participating in wastewater treatment.

The purpose of this review article is to investigate the potential of MFCs in the field of sustainable wastewater treatment and renewable energy production. More specifically, the following will be discussed: (I) the role of MFCs in the remediation of environmental pollutants, (II) exploring the use of MFCs for treating wastewater, (III) analyzing the working mechanism and routes of various types of MFCs and their construction features as depicted in the literature, and (IV) proposing some strategies to improve MFC performance.

## 2. Architectural Design for MFC Construction

**Figure 1:**
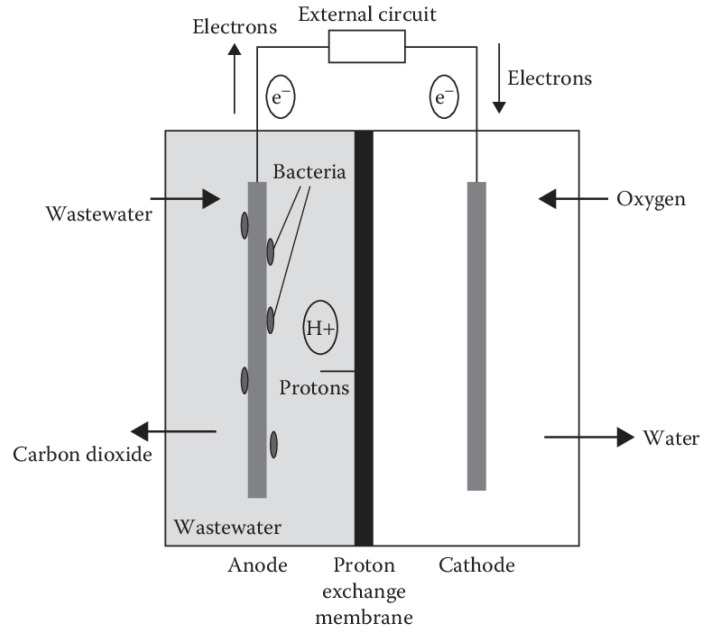
Schematic diagram of a typical dual chamber microbial fuel cell.

Figure 1 depicts a schematic of a typical MFC that generates energy, which shows that the cell contains an anodic chamber, cathodic chamber, and proton exchange membrane (PEM). The anodic chamber of the MFC is kept at an anaerobic condition where the microorganisms take part in the generation of electrons and protons. As an oxidation byproduct, carbon dioxide is created. The anode absorbs the electrons, which are subsequently delivered to the cathode. The electrons are passed via an external circuit. On the other hand, protons reach the cathodic chamber by passing through the PEM or a salt bridge. They react with O_2_, which commonly acts as the electron acceptor to generate water in the cathodic chamber [13]. Continuous electric current production is made feasible by separating microorganisms from oxygen or another electron acceptor besides the anode. To ensure this condition, the anodic chamber is made anaerobic [14]. Changes in the kinds of microorganisms, membranes, anodic surface modification, bacterial gene alterations, and other methods have been tried in the past to boost the generating capacity of electricity and the efficacy of the system in treating wastewater [15,16]. 

The performance of MFCs is highly dependent on their construction features. Other parameters impacting the performance of MFCs include the substrate supply, temperature, the microbial species or community employed, the anolyte composition, anode, cathode, and separator materials of the MFC. The three most critical components of an MFC are the anode, cathode, and PEM. Several anode and cathode materials have been utilized to increase or alter the capacity and performance of MFCs over time. 

### 2.1. Anode Materials

**Figure 2:**
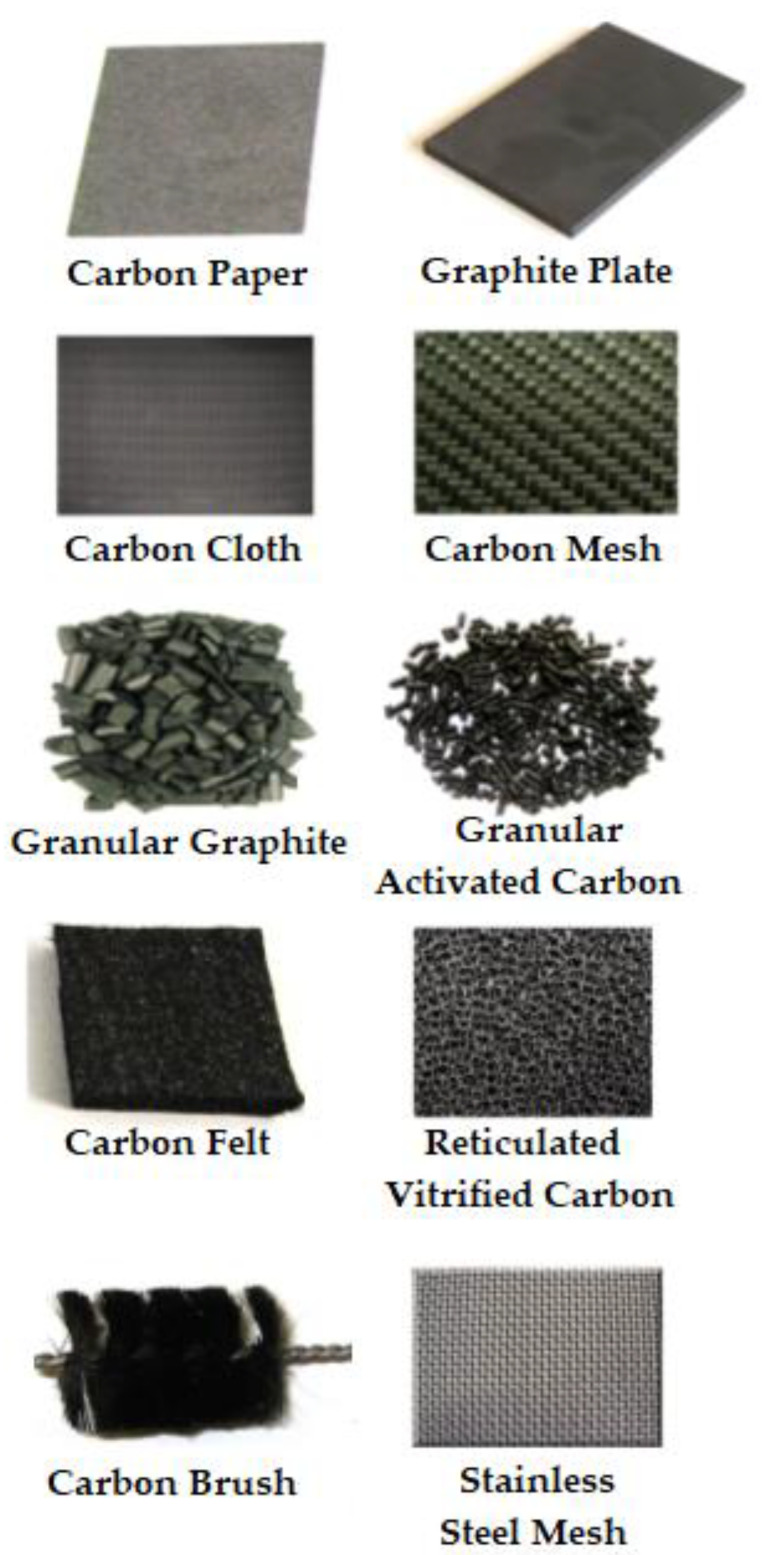
Different anode materials used for microbial fuel cells [adapted and modified from reference [22] with permission, 2023, Elsevier.

The selection of appropriate anode material is very significant for the performance and efficiency of MFCs. In a polarized electrical device, the anode is the electrode via which current enters from the external circuit. Oxidation takes place at the anode. In the past decade, the design and fabrication of anode materials influencing the performance of MFCs has attracted attention through several studies. The anode material controls the adhesion of bacteria to its surface for stable biofilm formation. The formation of stable biofilm is necessary for efficient electron transfer. It should also be biocompatible with the electroactive microbes and show enhanced conductivity for the electrons. In addition, the anode material should have properties such as anti-corrosion, mechanical strength, and chemical stability [17]. In general, the surface roughness of the anode materials is increased to ensure bacteria adherence, allowing higher power density. Additionally, the enhanced surface roughness increases durability against swelling and decomposition due to the redox condition at the electrode. On the contrary, it can increase fouling, which can affect its applicability in the long term. As a result, the durability and insensitivity to changes in operating conditions and pH should be taken into consideration while selecting appropriate materials for anodes. Moreover, the increased porosity and surface area of the anode allows stable biofilm formation [18]. Other key factors to consider while selecting and designing an anode including the extent of mass transfer, growth of the biofilm, ohmic and activation losses, and redox reactions [19]. Among these factors, the biofilm’s formation and growth are dependent on the surface properties of an anode, while ohmic loss is dependent on the internal resistances of the material [20]. As a result, studies have been conducted to design anode materials considering the above factors to optimize the efficiency of MFCs. Throughout time, numerous sorts of materials have been utilized to manufacture anodes. Different carbonaceous materials are widely applied as the material for the anode in an MFC [21]. Additionally, some metals and metal oxides have also been used as the material for anodes. The anodes manufactured using different materials can vary in design, surface area, and other properties. The properties and applicability of different anode materials are covered in the preceding part of this section [22]. Figure 2 shows different materials used for the construction of anodes in MFCs.

#### 2.1.1. Carbonaceous Anode

Carbon is the most predominantly applied anode material for MFCs due to its superior biocompatibility, excellent chemical and thermal stability, high conductivity, high mechanical strength, and inexpensive cost. The high surface area to volume ratio and the rough surface property of carbonaceous anodes provides more space and more favorable conditions for bacteria growth, resulting in better anode performance in MFCs. The surface properties such as large surface area to volume ratio and roughness allow enhanced bacterial growth, resulting in the efficient performance of the MFCs. Other desirable properties of carbonaceous anodes are their biocompatible and non-corrosive nature. These properties of carbonaceous anodes need to be explored more extensively than other metal/metal-oxide-based anodes [23]. In the last few decades, the application and development of carbon-based anodes have been through research and several studies. Different varieties of carbonaceous material, including carbon cloth, graphite rod, carbon plates, etc., have been used as anodes for MFCs. These carbon-based electrodes can be split into three categories: flat structure, packed structure, and brush structure. 

The most common carbon-based plane structured materials used for electrodes include carbon paper, carbon cloths, graphite sheets, and plates, etc. [24]. Among these materials, carbon paper is quite thin and a bit fragile. The advantage of carbon paper is that it allows easy connection of wires. In comparison, graphene sheets are more long-lasting than carbon paper. Graphite electrodes are found to be more efficient when the surface is roughened. However, they are too costly to be used in large-scale applications [25]. On the other hand, carbon fabrics are more favorable than sheets due to being more porous and allowing a large surface area. However, challenges are associated with its large expense [26]. Carbon mesh can be a favorable option in terms of cost compared with the sheet or cloths, producing larger power density [27]. Graphite rods, felt, and foams are used as anode material. Graphite rod is inexpensive, highly conductive and stable. However, enhancing its surface area is difficult. In a study conducted by Chaudhuri et al. (2003), the electrodes made using graphite rod, felt, and foam were compared. It was found that graphite foam produced enhanced power density and cell biomass compared with the other types of anode materials [28]. Table S1 presents the cost of different 2D and 3D carbonaceous anode materials for MFCs [29].

Carbon-based electrodes can be used in packing form to enhance the surface area available for bacterial growth. Granular or irregularly shaped packing can be used in the anode chamber of the MFC. Granular graphite can be used as packing material, which allows a large specific surface area. The packing bed is made conductive by stacking them close to each other. Granular activated carbon, carbon felt, small cubes of graphite, etc., can also be used as packing material for anodes. Graphite as a brush structure can also be a suitable electrode due to its large surface area, porosity, and current density. Logan et al. (2007) were the first to conduct MFC operations using brush anodes. In their study, the brushes were made using carbon fiber that was cut up to a certain length and coiled around non-conductive titanium wires. The brushed anodes were found to be more efficient than the conventional carbon paper anodes [30]. 

The advantages of carbon-based anodes include enhanced electrical conductivity, biocompatibility, and being relatively inexpensive. However, they suffer from their limited electrocatalytic activity. As a result, several modification strategies can be proposed to enhance their performance and optimize power output. Table S2 presents the MFC efficiency of carbon paper, carbon cloth, graphite plate, carbon mesh, carbon brush, etc., based on anodes.

#### 2.1.2. Carbon Nanotubes (CNTs) 

Carbon nanotubes are an excellent illustration of the use of nanotechnology. CNTs have the potential to be appropriate materials for anodes due to their large specific surface area, mechanical strength, stability, and conductivity [31]. Recently, conductive polymer/CNTs have captured the attention of several researchers. Researchers demonstrated that CNTs have the ability to enhance electron transfer of the anode and the effective surface area. In addition, polyaniline, a conductive polymer, protects microorganisms and enhances electrocatalytic activity [32]. Zou et al. (2008) constructed an MFC with a multi-walled CNT with carbon paper as the anode. The power density for the multi-walled CNT anode was found to be larger than that of the graphite anode. The enhanced efficiency was due to the presence of the carboxyl group on the surface. Owing to the large effective surface area and other surface properties, CNTs can improve the efficiency of MFCs [33,34]. The enhanced accessible effective surface area in groove openings and outside surface area of the CNT bundles allow it to be more efficient than conventional microporous adsorbent media. However, there are still certain challenges to overcome for large-scale applications, including clogging, higher operational cost, and the complex production process of CNTs [35,36].

#### 2.1.3. Graphene 

Graphene is a single-layer crystalline nanomaterial where the carbon atoms are arranged in a hexagonal lattice. In MFC operation, graphene has huge potential owing to its excellent electrical conductivity, high mechanical strength, large surface area, and biocompatibility [37,38]. The electron mobility of graphene is much higher than that of other carbon-based materials due to its structure [39]. As anode material, different forms of graphene, such as graphene oxide, modified graphene, reduced graphene oxide, etc., have been used due to their properties suitable for efficient MFC operation [40]. Zhang et al. (2011) were the first to introduce a graphene-based bioanode system for MFCs. The modified bioanode exhibited better performance than the conventional steel mesh anode. The enhanced electricity generation was due to the high surface area of the anode, allowing enhanced biofilm formation [41]. Additionally, the bioanode modified using graphene can allow enhanced mass transfer for the biofilm [42].

#### 2.1.4. Conductive Polymers 

Conductive polymers have gained attention as they can improve the performance of the MFC due to their excellent conductivity, enhanced bacterial adhesion, and increased biochemical activity [43]. Polypyrrole (PPy), Polyaniline (PANI), and other polymers have been used in MFCs as anodes. The conducting polymers are mostly aromatic with conjugated double bonds in their structure. PPy is the most widely utilized polymer in MFCs due to its increased stability and strong conductivity [44]. The synthesis procedure for PPy is also simple, and its surface properties can be varied easily to optimize its performance. Besides PPy, PANI has also received attention as a conducting polymer to be utilized in MFCs due to its biocompatibility, environmental durability, and low cost. However, the challenges for conductive polymers include structural instability, cathodic overpotential, and assembling of protons in the biofilm [45]. These challenges must be addressed for optimization between the cost and efficiency of MFCs using conductive polymers.

#### 2.1.5. Metal and Metal Oxide Anode 

Despite having higher conductivity compared with carbon-based anodes, metals are not being widely utilized as anodes for MFCs. The main factor to consider while selecting metals as the anode is their corrosiveness. The other challenge associated with metal anodes is their lack of biocompatibility. Among metals, stainless steel, titanium, platinum, etc., are utilized as base metals to be used as anodes. The roughened surface of the metal anodes can allow enhanced bacterial adherence [46]. However, some of the metals do not facilitate biofilm formation. Studies have shown that non-corrosive metals such as stainless steel could not produce increased power density due to poor bacterial adherence [21]. Other than this, different noble metals, such as platinum, titanium, gold, etc., have also shown improved performances. Noble metals can also act as catalysts for enhancing electron transfer. However, the main problem with their large-scale implementation is their expense [47]. The problems associated with pure metal anodes can be mitigated by using metal-oxide-based anodes, which can reduce the internal resistance and improve stable biofilm formation through the reduction in the toxic effect on the microbes. The metal-oxide-based nanoparticles can be used to form composites with carbon-based materials or conductive polymers for improvement of the efficiency of MFCs [21,47]. Table S3 summarizes the efficiency of the metals applied as anode materials in MFCs. Table 1 presents the advantages and disadvantages of different types of anode materials.

### 2.2. Cathode Type for MFCs

The selection of cathode type and suitable material is a significant factor in optimizing the cost and performance of MFCs. Cathodes account for almost 50% of the expense of MFCs [48]. The cathode materials impact the power density and electrochemical performance of the MFCs. In most cases, oxygen reduction reaction (ORR) occurs in the cathode, which is the rate-limiting reaction at the cathodes. The cathode selection is significant as it influences the ORR. The two main types of cathodes include air-cathodes and aqueous air-cathodes with or without catalysts. Platinum and titanium are the most widely used catalysts [47]. Another category of cathode that has been used is the biocathode, which has gained attention in recent times [15,47]. 

#### 2.2.1. Air-Cathodes and Aqueous Air-Cathodes

Air-cathodes and aqueous air-cathodes are widely used in the laboratory scale for MFCs. These cathodes often require catalysts. The air-cathode is directly exposed to air, allowing abundant oxygen availability. It also contains a layer of catalyst and supporting materials [49]. This type of cathode does not require any aeration and can achieve higher power density. Their simple structure has allowed its use in wastewater treatment [15]. On the other hand, aqueous air-cathodes are submerged in the electrolyte containing dissolved oxygen, which acts as the electron acceptor. Similar to air-cathodes, they can also be differentiated based on the presence of catalysts. The performance of this type of cathode is limited by the solubility of oxygen in the electrolyte.

The most common type of conductive materials used for these types of cathodes includes carbon cloth, paper, platinum mesh, etc. Moreover, there are some certain requirements for the binders that bind the catalysts to the electrode. The common binders include perfluorosulfonicfonic acid and poly(tetrafluoroethylene) [47,50]. Further studies are being conducted to obtain novel binder materials suitable for MFCs in terms of expense and performance. 

One of the drawbacks of using cathodes with catalysts is the high expense associated with it. This problem can be addressed by the use of carbon-based material with a large surface area, allowing more current density. Different materials, such as graphite, activated carbon, etc., have been utilized to attain more efficient performance for air-cathodes and aqueous air-cathodes [49,51].

#### 2.2.2. Biocathode

Biocathodes utilize aerobic bacteria, which can biochemically catalyze ORR and enhance the performance of MFCs. The performance of biocathodes can be higher than that of the abiotic cathode catalysts. The biocathode is more advantageous than conventional catalysts due to its lower expense, sustainable operation, and protection against catalyst poisoning. Biocathode formation requires support on which the bacteria forms biofilm. For the formation of biocathodes, stainless steel is often utilized. Carbon-based materials such as graphite felt, activated carbon, granular graphite, etc., are also used for biocathode formation [52,53,54,55]. The efficiency of biocathodes can be increased by providing an enlarged surface area for biofilm formation. The biocathode also enhances performance by reducing internal resistance [56]. The advantages of biocathodes have allowed them to gain further attention, and investigations are being conducted to find biocathodes that can improve the performance of MFCs at a reduced cost. Table 2 presents the advantages and disadvantages of different types of cathodes.

### 2.3. Membrane Materials 

Membranes are the components that physically divide the cathodic and anodic chambers. Membranes are one of the most crucial elements in the development of MFCs. Membranes allow the proton to transfer and stop the diffusion of oxygen. The membranes also help in chemical and ionic conjugation. The current density of a single-chambered MFC without a membrane is greater. However, oxygen and substrate diffusivity increase due to the absence of membranes, allowing the reduction in coulombic efficiency (CE). Thus, a membrane contributes to the long-term efficiency of MFCs. However, the challenges associated with membranes are their expense, fouling, and increase in internal resistance. Throughout the duration of MFC research, scientists attempted to determine the optimal membrane separator for MFCs. The membrane for an MFC should have properties such as lower cost, internal resistance, selectivity, chemical and mechanical stability, and fouling resistance [58]. 

#### 2.3.1. Ion Exchange Membrane

**Figure 3:**
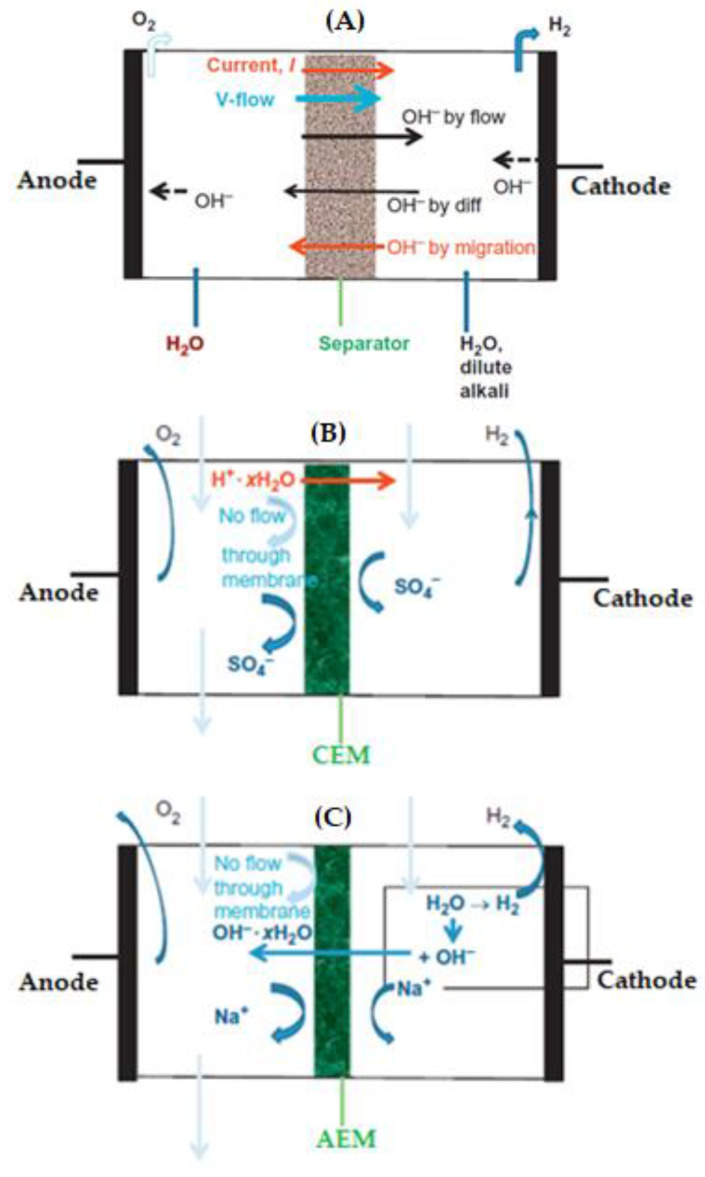
Ion transfer in the separator of a microbial fuel cell (**A**) and selective transfer of ions in a cation exchange membrane (**B**), and anion exchange membrane (**C**).

By their name, membranes that exchange ions are known as ion exchange membranes (IEMs). This technique was first introduced by the work of Ostwald in 1890. The ion transport phenomena of IEMs are illustrated in Figure 3A. With further improvements and investigations, a synthetic IEM was introduced for industrial applications. However, the obstacle associated with this type of membrane is the higher internal resistance for MFC application. Depending on the type of ion transport, IEMs can be divided into three categories, including Cation Exchange Membrane (CEM), Anion Exchange Membrane (AEM), and Bipolar Membrane (BPM) [58,59,60].

##### Cation Exchange Membrane (CEM)

CEMs allow the transport of protons and contain fixed negative charges (Figure 3B). The CEMs utilized for MFC application include Nafion, Zifrons, Hyflon, etc. These are applied due to their capability to conduct protons (Leong et al., 2013). Among the CEMs, Nafion is the most widely utilized due to its higher photon conductivity resulting from the presence of the sulphonate group [61]. 

##### Anion Exchange Membrane (AEM)

Anion exchange membranes (AEMs) allow the transport of negatively charged ions (Figure 3C). They contain positively charged ions. AEMs produce a larger current than that of CEMs in MFCs. The protons generated are consumed by the hydroxyl ion. This prevents an acidic environment in the anode chamber. As a result, the ion transport is increased at the anode, and the resistance at the cathode is decreased [62,63]. The conductivity of anions in AEMs is lower compared with the proton conductivity of CEMs due to the larger size of anions. The factors to consider in the utilization of AEMs include the retention of water, stability, and ionic conductivity. The challenges for the implementation of AEMs are mitigated by improving conductivity, the utilization of conductive polymer as binders, and optimization of the membrane assembly. Different inorganic fillers, such as TiO_2_, SiO_2_, bentonite, graphene, etc., have been utilized to retain water and improve conductivity [64].

##### Bipolar Membrane (BPM)

A BPM is a unique form of membrane made up of two monopole membranes. In contrast to monopole membranes, which only aid in the transport of selected ions, a bipolar membrane containing both an AEM and CEM offers effective transport of both H^+^ and OH^−^ ion over the membranes’ water-splitting interface [65]. However, the primary issue when employing such membranes is the pH gradient. Flat plate MFCs utilizing a BPM has been found to achieve a power density of 0.86 W/m^2^ [66]. 

#### 2.3.2. Porous Membrane

Porous membranes are the membranes where separation occurs on the basis of pore size. Some of the common examples are glass wools, UFMs (Ultra Filtration Membranes), and MFMs (Micro Filtration Membranes) [67]. These can be used as an alternative to traditional membranes in order to save costs. Low-cost glass wool may be used as a separator in single-chambered MFCs, which are extremely cost-effective for energy and environmental applications [68]. UFMs are most commonly used in MFCs, and they are permeable to both positively and negatively charged ions. 

In general, porous membranes suffer the same issues as membrane-free technologies. In addition to oxygen, the permeability of the membranes sometimes allows larger molecules to get through. The only advantageous characteristic of porous membrane is its reduced internal resistance, although this advantage is short-lived owing to biofouling. These challenges must be addressed to ensure the efficient utilization in MFCs [58]. Table 3 presents the advantages and disadvantages of different types of membrane materials.

### 2.4. Membrane Electrode Assemblies

The electrode (anode and cathode) layers can be associated with the membranes to produce membrane electrode assembly in an arrangement that reduces both the electrode space and ohmic resistance. The linkage between anode and membrane allows several limitations for both the membrane and anode. In membrane electrode assembly, there are IEMs, catalyst binders, and catalysts later in the cathode chamber. The cathode contains a carbon catalyst, which allows a large surface area. PTFE or PVDF can be used as a binder for the catalyst. The assembly of the cell can affect the performance of the MFC [13].

In MFCs, a salt bridge can be used. A salt bridge is assembled using a glass tube containing a saturated solution of KCl and phosphate buffer. The objective of the salt bridge is the conduction of ions and prevention of the accumulation. The separation of liquids is achieved by Agar. This makes oxygen diffusion difficult to predict for agar salt bridge when it is used in MDC [72]. Despite this, it can produce reduced power density and higher internal resistance. The power density can be increased by enhancing the surface area. On the other hand, the reduction in internal resistance can be achieved by reducing the electrode spacing. The overall efficiency of an MFC is dependent on microbial activity, construction, and arrangement of the cell. Overall, the generation of energy is controlled by the catalytic effect of the microbes, anode and cathode performance, and the transfer of protons [73].

Different types of configurations of MFCs have been introduced to optimize their operational efficiency, including single-chamber, double-chamber, and stacked MFCs.

#### 2.4.1. Single-Chamber MFCs

**Figure 4:**
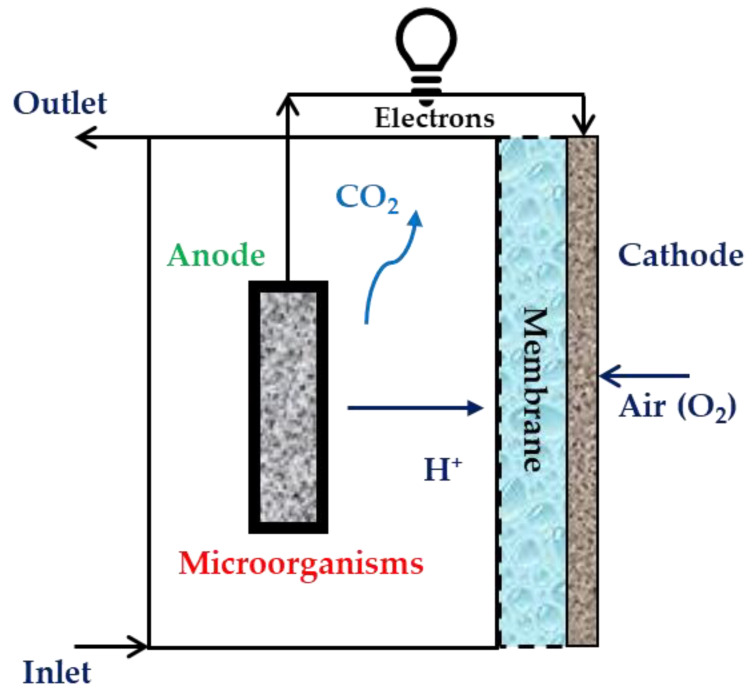
Schematic of a single-chamber MFC (with membrane).

Single-chamber MFC configuration is relatively more straightforward than double-chamber configuration. It consists of an anode in an anodic chamber connected with an air-cathode exposed to air [74]. In this configuration, the electron, after anaerobic digestion at the anode, is transferred to the porous cathode through the external circuit, where oxygen from air acts as the electron acceptor. The position of the IEM is close to the inner surface of the cathode. The membrane and porous cathode allow proton transfer to the cathode and prevent oxygen diffusion to the anodic chamber [75]. This configuration is less expensive owing to the absence of a cathodic compartment. Moreover, it does not require sparging of oxygen through the catholyte. In addition, the single-chamber configuration has lower internal resistance, enhanced oxygen reduction at the cathode, and higher power output. However, there are some limitations to this arrangement for commercial applications. The volume of the chamber cannot be increased, keeping the electrode spacing small, which decreases the power density when the arrangement is scaled up. As a result, it is not suitable for wastewater treatment for large-scale application [11]. Figure 4 illustrates the working of a single-chamber MFC with a membrane.

In single-chamber MFCs without any membrane or separator, higher proton transfer with a large power density could be achieved [76,77]. This would lead to a reduction in expenses. However, the drawback will be the reduction in CE resulting from oxygen diffusion. The activity of the microbes can also decrease [78]. To address these challenges, electrode spacing could be increased. However, it would result in increasing internal resistance. As a result, separators are required to remedy these problems associated with the single-chamber MFC. The separator allows enhanced CE and lower oxygen diffusivity. The separator should also allow higher proton transfer. However, the difficulty that remains with separators is their additional cost. As a result, further attention is required to obtain novel separation technology to optimize the performance and expense of MFCs.

#### 2.4.2. Double-Chamber MFCs

**Figure 5:**
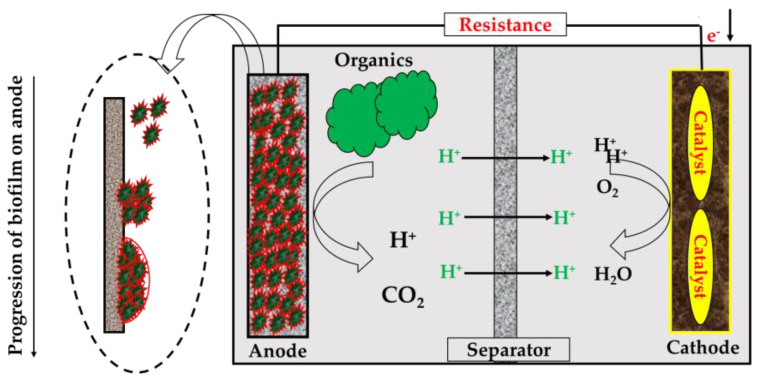
The working mechanism of a double-chamber microbial fuel cell.

Dual-chamber MFCs contain both anodic and cathodic chambers separated by membranes [79]. The microbes in the biofilm on the anode inside the anodic chamber perform anaerobic digestion of the substrate and generate electrons. These electrons reach the cathode through the external wire, and protons move toward the cathodic chamber through the membrane. The oxygen reduction reaction (ORR) occurs at the cathode. It requires a constant supply of oxygen in the cathodic compartment. The double-chamber MFCs have been modified and explored in different variations, including H-type, cylinder type, tubular type, flat plate type, etc. These modifications have been performed to enhance the operational efficiency for large-scale operations [74,80]. The H-type MFCs have reduced proton transfer due to a smaller surface area. As a result, they show lower power density. On the other hand, flat plate-type MFCs provide a larger membrane surface area, facilitating proton transfer. Moreover, it minimizes the distance between the electrodes, causing a reduction in internal resistance. Tubular MFCs have also emerged as a suitable option for large-scale operations [74]. The main advantage of double-chamber MFCs is that they can be applied for large-scale wastewater treatment to produce energy. The operation parameters that are required for scaled-up operation include maintaining suitable pH, the availability of sufficient O_2_, reduction in internal resistance, and addition of electron mediators [75]. Figure 5 depicts the working mechanism of a typical double-chamber MFC.

#### 2.4.3. Stacked MFCs

Another configuration that is being explored is a stacked MFC. In this configuration, the MFCs are connected in a series or parallel arrangement. This is being conducted to enhance power output and pollutant removal from wastewater [11,79]. The series connection allows the increase in voltage and reduction in treatment time. On the other hand, parallel connection increases both current and power density. The MFCs can also be connected vertically or horizontally [75]. The main limitations of this configuration are voltage reversal and high capital cost. If these issues are resolved, it can be a promising alternative for large-scale simultaneous wastewater treatment and power generation [74].

## 3. Role of MFCs in the Wastewater Treatment Process

**Figure 6:**
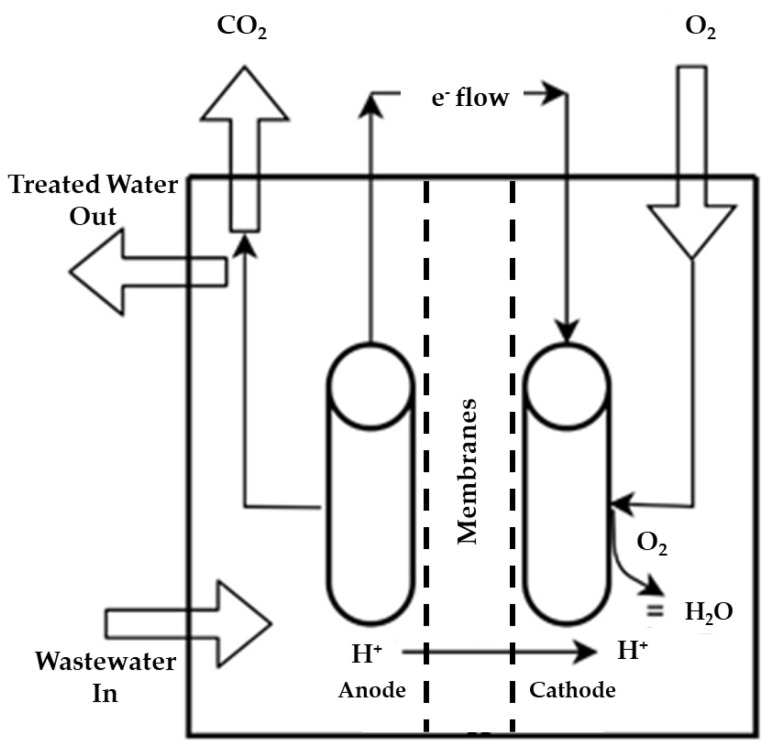
A schematic diagram of wastewater treatment in an MFC.

The MFC extracts a wide range of contaminants from wastewater effectively. Different categories of pollutants, e.g., petroleum hydrocarbons, organic matter, fats/oil, pesticides, dyes, etc., can be degraded by MFC technology [75]. Municipal, textile, tannery, distillery, and petroleum-based wastewater can be processed by MFCs in an efficient way. The chronological development of MFCs has played a significant role in wastewater treatment coupled with bioenergy development. The sustainability perspective of MFC-based wastewater treatment techniques combines the different prospects of MFCs, such as (I) the production of electricity via substrate (pollutant) degradation, (II) the aeration requirement being significantly reduced compared with the conventional activated sludge process, (III) operability at comparatively low temperatures, (IV) generation of fewer amounts of sludge associated with anaerobic digestion operation, and (V) can be accessible to areas with a low supply of electricity. Figure 6 shows a wastewater treatment process using MFCs. In the subsequent sub-section, different aspects of wastewater treatment by MFCs are discussed. 

### 3.1. Removal of Organic Compounds via MFC Treatment

The removal of organic substances by MFCs is accomplished through a biological process called biodegradation. The most important factors that affect the performance of MFCs are the electroactive microbial communities for the degradation of pollutants because of different metabolic exchanges among them [81]. MFCs convert organic and inorganic materials into current by using electrochemically active bacteria as catalysts [82]. Some bacteria have the capacity to transfer electrons from substrate to anode via endogenous mediators, nanowires, and pilis, as well as other microbial metabolites. These microorganisms are referred to as electrogenesis bacteria [83]. Inoculums of MFCs typically contain a variety of exoelectrogens, such as *Shewanella* sp., *Geobacter* sp., and *Rhodoferax* sp. [84]. 

The organic chemicals in wastewater are oxidized by microorganisms in the anode compartment of an MFC, converting them to less complex compounds such as carbon dioxide and water. By receiving the electrons produced by these oxidation events, the cathode compartment generates electricity. The primary factor in determining the cell potential is the metabolic route of the microorganism and the subsequent potential of the anode. In MFCs, bacterial catabolism is the rate-limiting process [85]. The stability and power output of MFCs is ultimately determined by these metabolic interactions. Consequently, an analysis of the microbial community structure has the advantage of improving the performance of MFCs [86]. In addition to producing power, the biodegradation process cleans the water by removing impurities from the wastewater, which can be reused or released back into the environment, making it an environmentally friendly and sustainable treatment solution. The reaction associated with MFC can be presented below:

Anodic reaction: CH_3_COO^−^ + H_2_O → 2CO_2_ + 2H^+^ + 8e^−^

Cathodic reaction: O_2_ + 4e^−^ + 4H^+^ → 2H_2_O

Overall reaction: ½ C_6_ H _12_O_6_ + H_2_ O + 3 O_2_ →4 CO_2_ +4 H_2_O

MFCs can use a variety of biodegradable organic molecules from landfill leachates, industrial waste, food waste, municipal wastewater, dairy waste, and other sources [87]. Additionally, MFCs can treat wastewater with a wide COD range, reducing both energy use and greenhouse gas emissions [88]. It has been determined that converting waste directly into high-value energy, clean electricity, or chemical goods is a superior solution for solving the excess sludge and energy problems in traditional wastewater treatment systems [89,90]. MFCs have been used in order to produce energy from different wastewater sources e.g., wastewater from food and food preparation industries, vegetable oil-based wastewater, refinery wastewater, fish handling wastewater, dairy wastewater, slaughterhouse wastewater, cautious cotton industry wastewater, animals and petrochemical endeavors, as well as from the wastewater of ripe squeezed apples. Dairy effluent can be a suitable substrate for MFC treatment due to the presence of a high amount of natural biodegradable compounds and nutrients [91]. From pure chemicals to complicated ones, MFCs have been successfully used in the treatment of organic chemicals such as glucose, acetate, butyrate [92], cysteine [93], proteins [94], and lignocellulose [95]. Lu et al. (2007) created an MFC framework with a 20 L capacity for processing wastewater from bottling operations. The maximum COD removal was 94.6%, with a flow rate of 1 mL/min and 313 h of water-powered maintenance. According to Firdous et al. (2018) [96], The efficiency of MFCs was increased by raising the temperature and duration. The highest COD removal rate (80–90%) at a high voltage (5839 mV) was attained with vegetable oil wastewater treated in a two-chamber MFC at 350 °C and a temperature of 80–90 °C. Using the maximum power density of 45 mW/m^2^, 86% removal of Chemical Oxygen Demand (COD) and 83% removal of NH_4_^+^-N were achieved when treating swine wastewater in a dual-chambered MFC [97]. A COD removal efficiency of 63% at the Organic Loading Rate (OLR) of 1.4 kg COD/m^3^ × d, yielding a current density of 863 mA/m^2^ and a power of 0.198 W/kg COD removed at an external resistance of 100 Ω, was recorded for treating wastewater from the chemical industry [98]. Scott et al. (2007) presented a stable performance of MFCs using carbon cloth as anode and cathode for a peak power of 5 W/m^2^, in which platinized carbon cathode doubled the power density [99]. Most MFC designs reportedly have COD removal efficiency of between 80 and 95% when treating various types of wastewater, proving the effectiveness of MFCs as a wastewater treatment system [100]. The potential benefits of MFCs in the treatment of wastewater and the production of bioenergy depend on a number of variables, including substrate choice (the kind of wastewater), reactor architecture, biocatalysts, and physiological constraints. In order to achieve better results, it is essential to maintain the right conditions for wastewater treatment in MFCs. In Table 4, COD removal efficiency and power generation capacity for different MFC configurations have been demonstrated.

### 3.2. Treatment of Nitrogen and Suphides with MFCs

Nitrogen-rich effluents should be treated before discharging them into water bodies. Direct discharge of nitrogen-rich effluent from composting facilities, oil operations, landfills, and domesticated animals into the environment can lead to a variety of problems, including eutrophication of water bodies and methemoglobinemia in children [111]. Nitrogen may be present in these effluents in altered forms, including ammonium, alkali, nitrate, and nitrite.

The well-known nitrification–denitrification reaction is the traditional biological nitrogen removal process, although it is energy-, carbon-, and cost-intensive. Denitrification, or the dissimilatory reduction of nitrate to nitrogen gas, is a step in this process that involves the aerobic oxidation of ammonia to nitrite (NO_2_) and nitrate (NO_3_) [112]. The idea behind the MFC depends on the fact that electroactive bacteria in the anode chamber oxidize organic substrates during the carbon and nitrogen removal process, generating electrons in the process. The generated electrons are then moved via an external circuit from the anode to the cathode. Then, by absorbing the electrons, bacteria in the cathode chamber denitrify the nitrate [113]. The reactions in the cathode are described below [114]:

NO_3_^−^ + 2 e^−^ + 2 H^+^ → NO_2_^−^ + H_2_O

NO_2_^−^ + e^−^ + 2 H^+^ → NO + H_2_O

NO + e^−^ + H^+^ → 1/2 N_2_O + 1/2 H_2_O

1/2 N_2_O +e^−^ + H^+^ → 1/2 N_2_ + 1/2 H_2_O

Nitrogen can be transformed or removed according to several experiments utilizing air-cathode MFCs. During operation for 100 h, an air-cathode MFC fed with swine wastewater had an ammonium removal rate of 83% [115]. Another investigation on decentralized wastewater treatment systems shows that MFCs can reduce total nitrogen by 60–80% [116]. You et al. (2009) observed that inoculating the nitrifying mixed consortia caused ammonium oxidation in the oxic biocathode [117]. In a biocathode MFC, simultaneous aerobic nitrification lowered the footprint of power production. Moreover, the nitrification process generated extra protons, which helped with energy generation by lowering ohmic resistance and keeping pH levels balanced. As opposed to abiotic methods, biocathodes have the benefit of preventing the buildup of intermediate mixtures such as nitrate from preventing contact. Both nitrate and nitrite can be employed as terminal electron acceptors by anodophilic bacteria; as a result, they are both oxidized in an active environment, either spontaneously or electrochemically. By using *Geobacter* sp., nitrate was physiologically converted to nitrite [118].

Park et al. (2017) showed an arrangement of five units of air-cathode MFCs in order to eliminate natural and nitrogen compounds from domestic wastewater at a Hydraulic Retention Time (HRT) of just 2.5 h, which achieved a removal rate of 85% of COD and 94 % of total nitrogen [119]. In another study, Yang at el. (2019) reported a single-chambered MFC augmented with wastewater, a consortium of Thauera-governed de-nitrifiers, that removed 90% COD, 98% alkalinity, and 95% of total nitrogen [120]. Nitrified wastewater can undergo denitrification by accepting electrons at the cathode. The cathode compartment produced a maximum power and current of 34.6 W/m^3^ and 133.3 A/m^3^ of cathode volume, respectively, while achieving removal rates of up to 2 kg COD/m^3^ × d and 0.41 kg NO_3_^−^—N/m^3^ × d in the anode and cathode compartments, respectively. It has been reported that the cathode can denitrify up to 0.146 kg NO^3^ —N/m^3^ × d. The denitrification system’s greatest power output was 8 W/m^3^ of cathode volume with a cell voltage of 0.214 V and a current density of 35 A/m3, respectively [121]. When a hybrid lab-scale MFC system (MFC combined with algal biofilm, AB-MFC) was employed in a batch mode, the efficiency of removing nutrients (TN 96%, TP 91.5%) from domestic wastewater rose. Compared with MFC (52.33 mW/m^2^), the Algae Bacteria MFC produced energy at a rate that was 18% greater [122]. In a recent study, it was found that using nitrifying–denitrifying bacteria, including Nitrosomonas, Clostridium, Pseudomonas, Arcobacter, and others, air-cathode MFCs could remove up to 95% TN, 91% COD, and 99% of ammonia [123]. Moreover, denitrification and energy production from the oxidation of organic molecules on a synthetic substrate has also been accomplished using marine bacterial communities [124]. In addition, the biocathode-based baffle reactor demonstrated an abiotic cathode to cathodic coulombic efficiency (CE) of 97.7 ± 1.9% and an autotrophic denitrification yield of 148.3 ± 1.4% of the net cathode volume. Another effective method has been developed for the recovery of nutrients, energy, and water from wastewater. It is known as an osmotic MFC. By boosting current output by 1.8 ± 0.1 A/m^2^, which increased ammonium removal from 40.7 ± 2.4% to 85.3 ± 3.5%., this approach changed the mobility of ammonium ions. The ammonium removal efficiency was increased to 55.2% from 6.5% by the water flow [125]. Wastewater containing ammonium and sulfide is converted into considerable energy (428.0 ± 26.2 C/batch cycle) by a three-chambered oxic-cathode and anoxic-cathode MFC with a similar anode. Wastewater was treated to remove and recover pollutants, including a nitrogen sulfide removal (206.5 ± 1.9 g S/m^3^ × d) and sulfur recovery efficiency of 28.9% (10.0 ± 1.3 g N) at a temperature of 300 °C, in addition to producing power. The elimination of this nitrogen related to the generation of electricity was regulated by the coulombic quantity at both cathodes [126]. Table 5 summarizes the nitrogen and sulfur removal efficiency and power generation capacity for different MFC configurations.

MFCs are also studied to remove sulfide from wastewater. Numerous industries emit sulfide, including petrochemical facilities, tanneries, and viscose rayon manufacturing lines [128]. A substance containing sulfide is toxic and has a negative impact on human health. The sulfate-rich wastewater treatment can be treated in three stages: (I) the reduction of sulfate, (II) the production of sulfide, and (III) the oxidation of sulfide. Due to the existence of a redox environment in microbial fuel cells, all of these may be integrated into a single phase within the anode chamber. These processes are catalyzed in the MFC via a mixed consortium of microorganisms functioning in concert for the treatment of sulfate-laden wastewater. Therefore, the injection of sulfate-reducing and sulfur-oxidizing bacteria into the anode chamber may be an energy-efficient method for treating sulfide-rich wastewater. In this process, sulfate is reduced by sulfate-reducing bacteria to hydrogen sulfide at the expense of available electron donors. Sulfur oxidizers then convert hydrogen sulfide to elemental sulfur, and sulfur precipitates at the anode. The sequence of reactions involved can be represented as follows:

Organic matter + sulfates → hydrogen sulfide + bicarbonates

Hydrogen sulfide → protons + electrons + sulfur

The sulfate-reducing bacteria named Desulfovibrio desulfuricans was used to inoculate a single-chamber air-cathode MFC [129]. As an electron acceptor in the process, the bacteria used lactate to convert sulfate to sulfide. Through the oxidation of sulfide to biogenic elemental sulfur, an additional 5100 mW/m^2^ of power density was generated. One study found a 63% increase in sulfate conversion to elemental sulfur by adding lactate to a dual-chamber microbial fuel cell as a terminal electron acceptor for sulfate reduction [130]. In contrast, 72% of elemental sulfur was recovered using acetate as the terminal electron acceptor instead of lactate. Since a range of physical, chemical, and biological pathways are involved in the treatment process, studies on microbial communities are essential for gaining a better understanding of the processes occurring in MFCs. Otherwise, biological activity might be attributed to the chemical events occurring in the MFC and vice versa, leading to erroneous conclusions regarding the redox reactions and the byproducts [112,113,114].

### 3.3. Treatment of Heavy-Metal-Rich Wastewater

Nowadays, one of the most significant environmental issues is heavy metal pollution. The treatment of heavy metals is particularly important due to their resistance, persistence in the environment, and toxicity [131]. Heavy metal pollution in wastewater refers to the presence of high levels of toxic metals such as lead, mercury, cadmium, and arsenic in wastewater that is generated by various industries, agricultural practices, and household activities. This pollution can adversely affect human health and the environment [132,133]. Different treatment processes, e.g., chemical precipitation, adsorption, ion exchange, etc., are commonly utilized to remove heavy metals from wastewater before it is released into the environment. However, much work has been focused on creating innovative clean methods for the recovery of metals in an effort to make the treatment of these metals both affordable and environmentally beneficial [134]. Microbial fuel cells can play a vital role in order to address the issue. The metabolic activities of microorganisms in a microbial fuel cell (MFC) can play a role in removing heavy metals from wastewater. The two main mechanisms by which this occurs are adsorption onto the cell surface and chemical precipitation.

Adsorption is a process in which heavy metals in the wastewater adhere to the surface of the microorganisms in the MFC. This occurs because the surface of the microorganisms has a positive charge, while the heavy metals have a negative charge. The two charges attract each other, causing the heavy metals to stick to the cell surface. On the other hand, chemical precipitation refers to the formation of insoluble compounds from heavy metals and other substances in wastewater. For example, heavy metals can form insoluble salts with sulfide or hydroxide ions that are produced by the metabolic activities of the microorganisms in the MFC. These salts are not soluble in water, so they precipitate out of the wastewater and can be removed with settling or filtration.

Although different metals were studied to be extracted using MFCs, the most successful studies were found with Cr removal [135]. The electrochemical reduction of Cr(VI) in MFCs utilizing graphite plate electrodes was initially investigated by Wang et al. (2008) [136]. At a concentration of 200 mg Cr(VI)/L, a maximum power density of 150 mW/m^2^ was produced at a reduction rate of 0.67 g/m^3^/h. Gupta et al. (2017) tested for the first time the viability of using a cheap electrode in a mediator-less DCMFC made of alumina (AA)/nickel (Ni)-nanoparticles (NPs)-dispersed carbon nanofiber (CNF) in an effort to lower the operational costs of MFCs while improving the reduction rates of MFCs [137]. MFCs are also used to treat silver effluent. With starting concentrations ranging from 50 to 200 ppm, silver recovery efficiencies as high as 99.91–98.26% were achieved in a cost-effective MFC, with a maximum power density of 4.25 W/m2 after 8 h of operation. Heijne et al. (2010) examined copper recovery in a DCMFC by dividing the two chambers with a bipolar membrane. With a maximum power density of 0.43 W/m^2^ produced under anaerobic circumstances in the cathodic compartment, removal efficiencies of around 99.88% were achieved [138]. Nearly 40% of Pt was recovered, and the recovery efficiency increased with longer reaction times [139].

### 3.4. Organic Dye-Based Pollutant Removal through MFCs

Today, the discharge of wastewater containing dyes is seen as a significant ecological concern. Dyes are often persistent organic pollutants that do not easily break down and can accumulate in the environment, leading to long-term pollution. Dye effluent is an example of highly colored wastewater that is biodegradation-resistant. Up to 70% of all produced textile dyes are azo dyes, which are distinguished by nitrogen-to-nitrogen double bonds (–N=N–) as the chromophore in the molecular structure [140]. Microbial fuel cells, on the other hand, present a potential approach to the removal of dyes from wastewater. The organic materials in wastewater, including colors, can be oxidized by the bacteria in the anode compartment of a microbial fuel cell, dissolving it into simpler compounds. Few reports have been made on dye decolorization in the MFC cathode, while the majority of studies have concentrated on dye decolorization in the anode. The co-substrate was typically oxidized (electron donor) and sent a fraction of electrons to the electrochemically active bacteria gathered on the anode, which then passed via an external circuit and produced current [141]. The relatively low electron transfer between the bacterial catalysts and the anode, which results in high internal resistance and insufficient power generation, is a significant barrier to the use of MFCs for dye decolorization. Therefore, it is crucial to understand the metabolic bottlenecks, dynamics of the electrochemically active bacterial (EAB) communities in the biofilm, and how the bacterial species can contribute to extracellular electron transfer if we want to increase MFC performance. Many of these microorganisms have the capacity to transmit electrons directly, which has attracted a lot of interest for the use of MFCs in dye degradation [141]. In mediator-less MFC systems, it has been demonstrated that pure strains of *Proteus hauseri* ZMd44 [142] and *Pseudomonas aeruginosa* [143] concurrently produce electricity and degrade dyes. In the Pseudomonas-catalyzed MFC, which is capable of generating pyocyanin and numerous other electron-shuttling chemicals, various azo dyes, including methyl orange (MO), Congo red, reactive blue 172 (RB), reactive yellow 145, and reactive red 2 were examined [143]. In mediator-less MFC systems and dye removal, several isolates, including *Geobacter sulfurreducens* and Beta Proteobacteria, have also been demonstrated to produce electricity [144].

As shown in Table 6 below, MFCs that use mixed bacterial cultures offer some significant advantages over MFCs powered by anoxic pure cultures, including a broader substrate specificity and greater power output. Table 6 summarizes different dye removal efficiencies and power generation capacities for different MFC configurations.

Han et al. (2017) showed that within 5 h approximately 98% of methylene blue was eliminated in the cathode chamber while simultaneously generating 36.56 mW/m^2^ of bioelectricity. The maximal power density produced by the complete breakdown of methyl orange to amines was 34.77 mW/m^2^. The maximum power output decreased significantly from 35 mW/m^2^ to 1.5 mW/m^2^ from pH 3 to pH 9, and the rate of decolorization was found to be considerably pH-dependent. Recently, a single-chamber arrangement with a microalgal biocathode was utilized to treat the real dye effluent. With a decolorization efficiency of 84–93%, the MFC was able to successfully remove around 98 percent of COD and zinc [150]. In the past, it was claimed that a membrane-less MFC could generate 1.7 W/m^3^ of energy while removing 73% and 77% of color from dye wastewater at the anode and cathode, respectively. The effluent was pronounced non-toxic within 24 h, and after a 48 h retention period, 71% of COD at the anode and 76% of COD at the cathode had been eliminated [151]. The degradation of the diazo dye Acid red 114 was investigated in a dual-chamber MFC bioanode with an SRB consortium controlled by *Desulfovibrio* sp. With 258 mW/m^2^ of simultaneously generated energy, almost 85% of COD and dye at an initial concentration of 100 mg/L were eliminated [152]. Similar to biodegradation, a complex mechanism that includes reduction, oxidation, and an association of enzymes is responsible for the breakdown of dyes in MFCs. This mechanism is reliant on the microbial community in the anode/cathode chamber. The only distinction from other bioreactors is the presence of PEM/CEM and the electrical circuit, which comprises the anode, cathode, and external load. This leads to a higher removal efficiency. However, a number of variables, including the kind and quantity of the dyes, the environment in the anode compartment, and the makeup of the bacteria, can affect how well microbial fuel cells remove dyes.

## 4. Limitation of Microbial Fuel Cells

Over the decades, the sequential improvements in MFC technology provided several advantages regarding simultaneous energy generation and wastewater treatment. However, certain limitations have hindered the widespread acceptability of MFC technology in industrial-scale applications. These challenges need to be addressed to overcome the limited adoption of this technology. The major limitations of MFC techniques are low power output, voltage instability, high internal resistance, mass transport loss, and biofouling. In order to run the system uninterruptedly, a stable voltage is required. However, the production of voltage is comparatively low in MFCs, and the stability of production is quite uncertain. Moreover, the operational performance of MFCs can be highly affected by the variation in the composition of the microorganisms, type, concentration of the organic matter in the influent system, and environmental conditions that lead to an MFC system with instability and reduced performance. These factors may lead to the power inadequacy to run an MFC system and make it challenging to use them for large-scale applications [153].

The main challenge associated with MFCs is their higher capital expenditure (CAPEX). Large CAPEX is mainly due to expensive IEM materials, electrodes, and catalysts. The CAPEX for MFCs is around 30 times that of conventional wastewater treatment technologies [11,154]. However, the operational cost for MFCs is lower due to the generation of electricity and less sludge production, eliminating the necessity for sludge treatment. With regard to energy generation, the power output of MFCs is relatively low compared with the theoretical limit. There are several sources of energy loss in the MFCs. There are losses due to the resistance offered by the conducting materials, separation media, and electrolytes. In addition, activation barrier loss at the electrode surface and mass transfer resistance of the chemical species at the electrodes contribute to additional energy loss [155]. As a result, there are scopes of improvement in power output. Moreover, the power output does not increase proportionately as the capacity of MFCs is scaled up. The lower power output is also associated with COD removal efficiency. The energy loss due to internal resistance can be minimized by reducing the distance between the electrodes. However, the reduction in electrode spacing can cause crossover of species between the anode and cathode chamber. Therefore, the membranes should be modified for closely spaced electrode arrangement [156]. The scale-up of the MFCs can be achieved by increasing the volume of the anodic chamber or stacking up multiple modular MFCs. However, there are several design and operational complications associated with these arrangements. The main issues for stacked MFC arrangement are voltage reversal, parasitic losses, and mass transfer hindrances [157]. The power output as well as CAPEX can be optimized by employing several strategies for the enhancement of anode and cathode performance, modification of membranes, and prevention of voltage reversal for stacked MFC arrangement [157].

Several studies reported that the performance of an MFC system, e.g., generation of electricity, wastewater treatment, etc., is critically dependent on different factors, e.g., (I) pH, (II) electrode materials, (III) resistance of the system, (IV) type and concentration of the electrolyte, and (V) concentration of dissolved oxygen in the cathode site [158]. Malfunctions in any of these parameters may lead to the low or non-efficiency of an MFC system [158]. The continuous voltage generation is hindered by the mass transport of oxidants from anodic to cathodic apartments. The inefficient diffusion of oxidants via membranes to the cathodic apartment creates reduced electron intake/rejection due to the saturation of oxidants in the anodic part, which results in an unstable voltage production [159,160]. Moreover, MFCs are highly influenced by the factors that are correlated to the growth of microorganisms. The electron transfer from the cathode to the anode is limited by the biofilm and kinetics of the microbes. This can be controlled by the usage of bacteria such as Geobacter or other genetically modified microbes [11]. Seasonal factors, e.g., temperature, humidity, etc., influence the performance of MFCs. In low-temperature seasons and regions, the growth of microbes is influenced by suppressing the metabolism that tends to decrease in electron excretion [153]. One of the most critical issues in MFC operation is the biofouling of a membrane that reduces the efficiency of the proton transfer rate in the cell. Due to biofouling, a periodical physical or chemical cleaning process is required. After certain periods of MFC operation, the membranes need to be replaced, which is very cost-intensive. There can be other factors related to biofilm that can influence the performance of MFCs. The performance of MFCs can be reduced by inactivating the electrocatalyst through salt generation and excessive biofilm growth, which results in dead cells or polymeric substances near the electrode surface, isolating it from electrochemically active bacteria. The approach that can be used for the control of biofilm is the application of a magnetic field to the microorganisms. It can increase or decrease microbial activity depending on the magnitude of the magnetic field [161]. Therefore, to improve the acceptability and feasibility of MFC systems, the limitations need to be addressed, which requires further research in this field.

## 5. Future Perspective and Recommendations to Improve Microbial Fuel Cell Performance

In recent times, MFCs have been introduced as a self-sustaining energy production and wastewater treatment technology [75]. However, the limitations associated with MFCs hinder their penetration at the industrial scale. In MFCs, the kinetics of microorganisms, development of optimized biofilm, and efficient electron transference between catalyst and electrodes have to be enhanced to supply a stable voltage and high power output, especially when waste substrates are used [162]. Therefore, it is necessary to develop highly efficient anodes for MFCs to facilitate the above-mentioned processes [15]. On the other hand, the cathodic ORR is crucial for MFCs. Due to the low kinetics and high overpotential of cathodic ORR, it controls MFCs performance to an extent [163]. Noble materials, e.g., platinum (Pt), offer high catalytic activity; however, the cost of Pt is very high, which is a major barrier to implement MFCs at the industrial-scale. Therefore, the search for other feasible catalysts should be accelerated to reduce the cost of MFC systems [164,165]. In terms of scaling up the MFC system, the selection of suitable separators often plays an important role in overall MFC design [69]. Separator-less MFCs have been developed recently. However, due to the uncontrolled diffusion phenomena within anode and cathode compartments in separator-less MFC systems, the energy generation and wastewater treatment efficiencies in these systems are very low compared with the conventional separator-based configurations. The traditional Nafion-based separator is costly and has O_2_ crossover issues; to solve these, CEM and AEM are already being used. More research should be concentrated on bipolar membranes, glass fibers, ultrafiltration membranes, and porous fabrics materials to enhance the ions’ diffusion [166]. In addition, MFC technology is sensitive to different operation factors e.g., substrate (waste product or carbon-source) concentration, ion species, pH, and temperature. The sensitivity level of MFCs for these parameters should be exploited for sensing purposes with online mode operation [167]. Although there has been ongoing research on the optimized design of MFCs to improve their performance, most of the studies are still focused on the MFC reactor configurations and on operational conditions. However, research on types of microorganisms and electrode materials is very limited. For the commercialization and industrial applications of MFCs, the optimization of microorganisms and the invention of novel electrodes which will provide a promising option for cost-effective bioelectricity generation through MFCs are required [168]. It can be seen that several units and processes associated with MFCs are being subjected to intensive research in order to improve their performance, with the ultimate objective of bringing this technology into the market for real implementation and commercialization. Due to the huge scope and broader market of MFCs, the research should not be confined to only two aspects (e.g., electricity generation and wastewater treatment) of MFCs; future research efforts should be focused on the development of hybrid MFC systems for biohydrogen and chemical production for making MFCs the best-suited technology with sustainable energy, chemical, and hydrogen production.

## 6. Conclusions

MFCs have become an efficient technology for self-sustainable wastewater treatment and have been extensively utilized in wastewater treatment. The organic materials in wastewater, including dyes and other organic pollutants, can be oxidized by the bacteria in the anode compartment of a microbial fuel cell, dissolving it into simpler compounds. There are several parts in designing a fully operational MFC. The architectural design of an MFC cell starts with the selection of anode, cathode, and membrane material selection and then the cell design. Carbonaceous materials, e.g., graphene, CNTs, metals, and metal oxides, have been studied and utilized for the preparation of anodes. Air and aqueous cathodes are widely used cathodes; recently, biocathodes have been utilized in MFCs. For membranes, IEM, e.g., CEM, AEM, bipolar, and porous membranes, are utilized. Cell assembly is an important design factor to operate MFCs efficiently. The anodes of MFCs have to be characterized by biocompatibility, surface area, porosity, stability, durability, cost, and availability. Moreover, the cathode of an MFC has to show intrinsic catalytic reactivity and appropriate binding strength. The proton transfer coefficient, selectivity, low oxygen diffusivity, antifouling properties, stability, and durability are the factors that regulate the membrane efficiency in MFCs. The conventional three-stage sulfate reduction can be treated in a redox environment in microbial fuel cells within the anode chamber. However, due to the limitations, e.g., low power output, voltage instability, high internal resistance, mass transport loss, and biofouling, the adoption of MFCs has been hindered at the industrial scale. Future research is needed to address these limitations, which can enhance the acceptability of the MFC technology in commercial self-sustaining wastewater treatments.

## Figures and Tables

**Table 1:** Advantages and disadvantages of different types of anode materials [21,35,36,39,44,45,47].

Anode Materials	Advantages	Disadvantages
Carbonaceous anode	High conductivity High stability Biocompatibility	Limited electrocatalytic activity Low power density
Carbon nanotube (CNT)	Large surface area High mechanical strength Stability Electrical conductivity	Clogging High operational cost Complex synthesis procedure
Graphene	Excellent electrical conductivity High mechanical strength Large surface area Biocompatibility High electron mobility	Complex synthesis procedure
Conductive polymer	Excellent conductivity Better bacterial adhesion Enhanced biochemical activity	Accumulation of proton biofilm Cathodic overpotential Structural instability
Metal	Expensive noble metals High conductivity	Poor biocompatibility Corrosiveness Low surface area
Metal oxide	Reduction in internal resistance Improved biocompatibility	Expensive for large-scale implementation

**Table 2:** Advantages and disadvantages of different types of cathodes [22,49,57].

Cathode Type	Advantages	Disadvantages
Air-cathode and aqueous air-cathode	Simple structure Cathodes can be modified using cheap materials such as activated carbon or HNO_3_ to enhance performance Recycling of catholyte not required for air-cathode	Performance of aqueous air-cathode limited by the solubility of oxygen Oxygen crossover Use of catalyst can lead to additional cost Biofouling of the cathodes
Biocathodes	Inexpensive Sustainable Protection against catalyst poisoning Reduction in internal resistance	Lower power output Fluctuation of pH

**Table 3:** Advantages and disadvantages of different types of membrane materials [69,70,71].

Membranes	Advantages	Disadvantages
Cation exchange membrane	Lower ohmic resistance resulting in lower internal resistance High proton conductivity	pH splitting Oxygen crossover Biofouling resulting in a reduction in ionic conductivity
Anion exchange membrane	Useful for alkaline fuel cells Prevent pH splitting	Substrate crossover Biofouling on the cathode
Bipolar membrane	Effective for desalination Prevent proton accumulation in anodic chamber	Polarization can be increased through water splitting Higher polarization leads to increased internal resistance
Porous membrane	Inexpensive compared with IEMs Low internal resistance	Non-selective to ions Oxygen and substrate crossover Biofouling

**Table 4:** COD removal efficiency and power generation capacity for different MFC configurations.

Organic Pollutant Sources	MFC Configuration Systems	Hydraulic Retention Time (h)	Operational Conditions	COD Removal Efficiency (%)	Power Generation Density (mW/m^2^)	References
Complex wastewater contaminated with drugs, chemical intermediates, dye and dye intermediates, pesticides	Design: Dual chamber Volume: 0.75 L	--	pH: 7.82 COD (g/L): 12.1	῀62	65.82	[101]
Confectionery wastewater	Cathode: Air Design: Single chamber Volume: 0.9 L	--	pH: 7 COD (g/L): 1.0	>92	373	[102]
Acetate (40 mM)	Cathode: Air Design: Tubular Volume: 0.2 L	--	pH: 7 COD (g/L):	--	--	[103]
Diesel contaminated wastewater, range organics -C8 to C25	Design: Dual chamber Volume: 0.45 L	--	pH: 3 (Cathode) COD (g/L): 0.300 g Diesel/ L	82	32	[104]
Synthetic wastewater	Design: Double cell Volume: 0.300 L	96	pH: -- COD (g/L): 0.812 g/ L of glucose	85 (TOC)		[105]
Filtrated wastewater plus acetate (glucose or xylose)	Design: Dual cell Volume: 0.600 L	_	pH: 7.6 COD (g/L): 1.13 g/ L of glucose	_	130 ± 5 (for acetate)	[106]
Domestic wastewater and glucose	Design: Dual chamber mediator less Volume: 0.24 L	--	pH: 7 COD (g/L): 0.5–0.6	--	9.3	[107]
Landfill leachate	Design: Single chamber downflow Volume: 0.90 L	4.7	pH: 7COD (g/L): 0.468- 0.630 (BOD)	57% (BOD)	0.19	[108]
Dairy industry wastewater	Design: Catalyst and mediator-less membrane MFC		--	COD: 90.46%	621.13	[91]
Molasses wastewater	Design: Single and dual chamber MFC		--	COD 89–90%	7.9 + 2.56	[109]
Coking wastewater	Design: Single-chambered fluidized MFC		--	--	2.13 + 0.01	[110]

**Table 5:** Nitrogen and sulfur removal efficiency and power generation capacity for different MFC configurations.

Organic Pollutant Sources	MFC Configuration Systems	Pollutant Removal (%)	COD Removal Efficiency (%)	Power Generation Density (mW/m^2^)	References
Domestic wastewater	Five units of air-cathode MFC	Nitrogen—94%	85%	--	[126]
Wastewater, consortium of Thauera	Single-chambered MFC	Nitrogen—95%	90%	--	[120]
Wastewater	Three-chambered oxic-cathode and anoxic-cathode MFC	Sulfur—28.9%	--	428.0 ± 26.2	[126]
Sulphate-rich pollutant	Dual-chamber	Sulfur—63%	--	5100	[111]
Agricultural wastewater	MFCs with single-chamber air-cathode and two chamber aqueous cathode	Ammonia—83%	83	45	[97]
Beat sugar wastewater	Up-flow anaerobic sludge blanket reactor MFC	Sulfate—52.7%	53.2%	1410.2	[127]

**Table 6:** Different dye removal efficiencies and power generation capacities for different MFC configurations.

Type of Dye in Wastewater	MFC Configuration	Microbe Sources	Initial Concentration (mg/L)	Color Removal Efficiency (%)	Electricity Generation	References
Acid orange 7	Two equal rectangular Perspex frames	Microbial consortium	0.06	--	0.31 ± 0.03 W/m^3^	[145]
Diazo dye C.I. reactive blue 160 (RBu160)	Single-chamber MFC	Proteus hauseri ZMd44	450–600	--	197 W/m^2^	[146]
Methyl orange	Dual-chamber MFC	Anaerobic sludge from Gaobeidian wastewater treatment plant	10–20	73.4	--	[147]
Congo red	Air-cathode	Mixture of aerobic and anaerobic sludge from Liede municipal wastewater treatment plant	300	90	192 mW/m^2^	[148]
Thionine-based textile dye	Membrane-free air-cathode single-chamber MFCs	Proteus hauseri ZMd44	40	--	83.39 ± 0.28 m	[149]
Reactive brilliant red X-3B (ABRX3)	Microbial fuel cell coupled constructed wetland (CW-MFC)	Microbial fuel cell coupled constructed wetland (CW-MFC)	300	95.6	0.852	[144]

## Supplementary Materials

The following supporting information can be downloaded at: https://www.mdpi.com/article/10.3390/membranes13050490/s1, Table S1: Cost of different 2D and 3D carbonaceous anode materials for MFCs, Table S2: Various Carbon anode materials and their performances in MFCs; Table S3: Metal materials applied as anode MFC and their performance in MFCs.
