# Supplementary material for: Microbial Fuel Cell Construction Features and Application for Sustainable Wastewater Treatment
Source: Membranes (Basel). 2023 Apr 30;13(5):490. doi: 10.3390/membranes13050490 (PMC10223362; doi:10.3390/membranes13050490)
Supplement: Supplementary file 1 [file membranes-13-00490-s001.zip › membranes-2283634-supplementary.pdf]

Table S1: Cost of different 2D and 3D carbonaceous anode materials for MFCs

2D electrodes	Cost ($)/size (cm)	3D electrodes	Cost ($)/size (cm)
**Carbon paper**<br>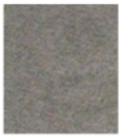	130/(40x40)	**Carbon felt**<br>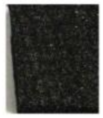	46/(40x40)
**Graphite plate**<br>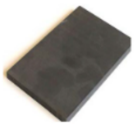	75/(30x30x0..5)	**Granular graphite**<br>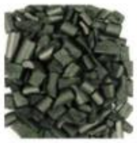	6-8/kg
**Carbon cloth**<br>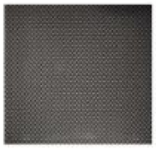	79/(45x40)	**Reticulated vitreous carbon**<br>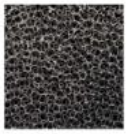	1.5/(1x1x2.5)
**Carbon mesh**<br>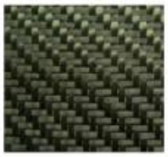	6-40/(100x100)	**Graphite rod**<br>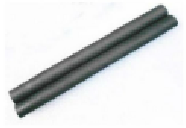	1-10/(1x20)

Table S2: Various Carbon anode materials and their performances in MFCs

**Anode Materials**	**Comments**	**MFCs Efficiency**
**Carbon paper**	Very thin, simple to join the wire, compact structure and surface, low specific area, high cost, lack of durability, and somewhat brittle.	600 mWm^{-2} based cathode projected area (bottle type MFC)<br>506 mWm^{-2} MFCs fed acetate and 305 mWm^{-2} fed with butyrate (based on anode area)
**Carbon cloth**	More flexible and far more permeable than carbon paper, thin and costly	1040 mWm^{-2} based cathode projected area (cube-shaped MFCs)<br>1640 mWm^{-2} of the MFC with a 200 mM phosphate buffer based on anode area

		46 mWm^{-2} based on total anode compartment
**Graphite plate**	Compact structure and a relatively smooth surface, low specific area, and high cost	3290 mWm^{-2} based on anode area 1078 mWm^{-2} based on anode area
**Carbon mesh**	Alternative to carbon paper and carbon fabric that is less costly, thin, and easily deformed.	The data for untreated carbon mesh was not reported; the stable voltages were produced from MFCs with all anodes except the untreated carbon mesh (CM)
**Granular graphite**	High specific area, porosities of the packed electrode are relatively low clogging after long-term running	48 and<br>38 Wmm^{-3} based on<br>total anode compartment, for feed streams based on acetate and glucose, respectively<br>175 Wmm^{-3} based on total anode compartment
**Graphite/ carbon felt**	Much thicker than the materials discussed previously, bacterial growth is more likely to be inhibited by the mass transfer of substrate and products on its inner surface, significant porosity, and high resistance.	356 Wmm^{-3} based on total anode compartment for carbon felt<br><br>386 Wmm^{-3} based on total anode compartment for graphite felt
**Reticulated vitreous carbon (RVC)**	Available with different pore sizes, conductive, and rigid but brittle material	39.4+3.0<br>Wmm^{-3} based on cathode area<br><br>170 mWm^{-2} based on anode area
**Carbon brush**	Ideal electrode with high surface area, high porosities, and efficient current collection. Clumping of fibers hindered bacterial access to the fiber surfaces as well as the diffusion of substrate into the brush interior	2400 mWm^{-2} based cathode projected area (cube-shaped MFC)<br>1430 mWm^{-2} based cathode projected area (bottle type MFC)

Table S3: Metal materials applied as anode MFC and their performance in MFCs

Anode	MFC efficiency

materials	
Cu	40 Wm^{-2}<br>based on anode area; the higher power density<br>must either be a calculation error or due solely to copper<br>corrosion with the solution used in the tests<br>2+0.5 mWm-2 based on anode area
Stainless steel	23 mWm^{-2} based on anode area Maximum current density of stainless steel electrode was around 2.4 Am-2 vs.1.1 Am-2 of graphite electrode<br>(constant potential chrono amperometry)<br>Maximal current densities reached under constant polarization at+100 mV vs. SCE: 3.1 Am^{-2} for plain stainless steel, 5.9 Am^{-2} for plain graphite, and 8.2 Am^{-2} for stainless steel grid, each of 25 cm^{-2} projected area
Ti	No date of the power density of Ti because daily current densities of Ti were far low +0.1 A m-2 than that of flat graphite, roughened graphite, and Pt-coated Ti with values between 2 and 2.5Am-2
Ag	0.8 mWm^{-2} of Al electrode vs. 1.8 mWm^{-2} carbon fiber cloth electrode
Al	0.004 mWm^{-2} of Al electrode vs. 1.8 mWm-2 carbon fiber cloth electrode
Ni	0.2 mWm^{-2} of Al electrode vs. 1.8 mWm^{-2} carbon fiber cloth electrode
Stainless steel	0.1 mWm^{-2} vs. 1.8 mWm^{-2} carbon fiber cloth electrode
Pt	78 Wm^{-2} for Pt nanoelectrode was connected to the wire covered with nanoparticle Ti vs. 35.08 W m-2 of simple electrode Pt to the simple Ti wire

N:B: None of these works are done by the authors of the paper. To get the source of the above mentioned works in Table S1,S2 and S3, please contact the corresponding author(s) of this article.
